# Immunohistochemical Staining Characteristics of Low-Grade Invasive Ductal Carcinoma Using the ADH5 Cocktail (CK5/14, P63, and CK7/18): A Potential Interpretative Pitfall †

**DOI:** 10.3390/diagnostics13182966

**Published:** 2023-09-15

**Authors:** Reham Al-Refai, Ahmed Bendari, Doaa Morrar, Sunder Sham, Layth Kataw, Azar Garajayev, Sabina Hajiyeva

**Affiliations:** 1 Department of Pathology and Laboratory Medicine, Northwell Health Lenox Hill Hospital, New York, NY 10075, USA; abendari@northwell.edu (A.B.); dua.m@hotmail.com (D.M.); ssham1@northwell.edu (S.S.); lkataw@northwell.edu (L.K.); shajiyeva@northwell.edu (S.H.); 2 Baku Health Center, Azerbaijan Medical University, Baku AZ1022, Azerbaijan; garajayevazer@gmail.com

**Keywords:** ADH5, multiplex stain pitfall, breast cancer, immunohistochemistry, diagnosis

## Abstract

**Background:** In our practice, the antibody cocktail ADH5 (CK5/14, p63, and CK7/18) helps with diagnostic challenges, such as identifying microinvasion and foci of invasive carcinoma, differentiating atypical ductal hyperplasia from hyperplasia of the usual type, and distinguishing basal phenotypes in triple-negative carcinomas. However, the ADH5 cocktail does have pitfalls and caveats. **Methods:** We describe our experience with the ADH5 cocktail of antibodies in breast pathology. Institutional knowledge and a literature search form our data sources. **Results:** We analyzed 44 cases. Four out of a total of 44 cases (9.1%)—two tubular carcinomas and two low-grade invasive breast carcinomas of no special type (ductal) with tubular features—showed an expected pattern of staining for ADH5 with a loss of brown (P63, CK5/14) staining around invasive glands and diffuse red (CK7/18) expression. Forty out of 44 (90.9%) cases showed an unexpected staining pattern (mixture of cytoplasmic brown and red). All 44 cases (100%) showed negative myoepithelial staining around invasive foci when separately stained for P63 and SMMH (Smooth Muscle Myosin Heavy). **Conclusions**: The unexpected staining pattern of ADH5 in low-grade invasive ductal carcinomas can be challenging to interpret in these lesions with low-grade cytology. The occurrence can cause confusion among users who employ multiplex stains, and it is important for users to be aware of this potential pitfall.

## 1. Introduction

Breast cancer is a highly prevalent cancer that primarily affects women and is the second leading cause of cancer-related deaths. While all women are at risk of developing breast cancer, the level of risk can vary among different populations. The lifetime risk of developing invasive breast cancer ranges from 3% for women with no identifiable risk factors to over 80% for women with certain risk factors, particularly those with highly penetrant germline mutations [1].

To address the importance of early detection, the American Cancer Society recommends that all women have the option to begin annual mammogram screenings starting at the age of 40. Expressly, women aged 45 to 54 are advised to undergo mammograms every year. Early identification is vital in improving survival rates after a breast cancer diagnosis. Advancements over the years have significantly improved screening approaches for detecting breast lesions. The routine use of mammograms and ultrasound and the emergence of newer imaging modalities such as magnetic resonance imaging (MRI) have enhanced the ability to detect and diagnose breast abnormalities [2].

The introduction of less invasive techniques in screening has led to the availability of smaller samples of lesional tissue for examination. This has necessitated focusing on microscopic features to differentiate among benign, atypical, and malignant breast lesions. Detecting cancers early may allow for more conservative surgical procedures, increasing the chances of a successful cure. However, diagnosing tiny tumors can be challenging due to their size and subtle characteristics, which may require specialized techniques and expertise [2].

The mammary glandular system (ducts and lobules) comprises an inner layer of epithelial cells and an outer layer of myoepithelial cells. This double-layered architecture is also observed in various proliferative conditions, including benign and atypical proliferations, sclerosing lesions, and in situ carcinomas. In contrast, invasive carcinomas, which are more aggressive and capable of spreading, lack a myoepithelial layer surrounding the glands [3]. However, it is important to note that the absence of a myoepithelium alone is not sufficient to diagnose invasive carcinoma, as it can also be lost in other benign conditions, such as microglandular adenosis, certain apocrine lesions, and specific types of in situ ductal carcinoma. Therefore, pathologists rely on the presence of an intact myoepithelial cell layer around cancerous cells as a critical diagnostic feature for differentiating in situ (non-invasive) from invasive carcinomas [3].

Pathologists employ various staining techniques to identify myoepithelial cells and determine their presence or absence. Smooth muscle actin is a widely used stain due to its sensitivity and specificity as a marker for myoepithelial cells. Additionally, specific cytokeratins (such as keratins 5, 7, 14, and 17) and the S100 protein can also stain myoepithelial cells, although they are not as specific or sensitive as smooth muscle actin [4]. Furthermore, a p53 homolog called p63 has been identified as a selective marker for human epithelial basal cells in different organs, including the skin, cervix, and prostate. In the context of breast cancer, p63 exhibits a high affinity for myoepithelial nuclei and demonstrates sensitivity and specificity in identifying them. Unlike other markers that stain cytoplasmic components, p63 specifically stains the nuclei of myoepithelial cells, making them easily visible under microscopic examination. This characteristic is particularly beneficial when examining fine-needle cytological preparations, where cytoplasmic fragments can sometimes interfere with accurate identification [5].

Grading invasive ductal carcinomas is essential for assessing breast cancer and estimating the degree to which a tumor resembles normal breast glands. Low-grade carcinomas, which closely resemble normal glands, tend to have a slower growth rate and a more favorable prognosis than poorly differentiated ones. One specific type of breast carcinoma with a particularly favorable prognosis is tubular carcinoma. Tubular carcinoma is characterized by the presence of small, round to ovoid, or angular glands and tubules with open lumens embedded within a fibrous or fibroelastotic desmoplastic stroma. The cells that line these neoplastic tubules typically have relatively uniform nuclei of small or intermediate size. To establish the diagnosis of tubular carcinoma, over 90% of the tumor must consist of tubules and glands lined by a single layer of neoplastic cells. This emphasis on the architectural pattern helps distinguish tubular carcinomas as low-grade carcinomas. Unlike benign conditions, the glands in tubular carcinoma lack a surrounding myoepithelium, which aids in their differentiation [6]. Low-grade carcinoma cells typically exhibit a luminal phenotype, meaning that they express cytokeratins 8 and 18 (CK8/18-positive) while lacking expression of cytokeratins 5 and 14 (CK5/14-negative). These immunohistochemical patterns provide added evidence to support the diagnosis of tubular carcinoma and help distinguish it from other breast lesions [6].

Breast core biopsy is the most common nonoperative method for diagnosing breast lesions. It involves the removal of small tissue samples using a specialized needle. Pathologists rely on these samples to perform a detailed microscopic examination and obtain diagnostic information. However, in some instances, pathologists need to provide a comprehensive diagnosis using limited tissue volumes. This necessitates careful analysis and optimization of the available tissue to extract as much diagnostic information as possible [6,7].

Efforts are continually being made to improve diagnostic techniques and refine the assessment of breast lesions. These advancements aim to enhance the accuracy of diagnoses while minimizing the amount of tissue required for analysis. By optimizing the diagnostic process, clinicians can effectively guide treatment decisions and provide patients with tailored care based on the specific characteristics of their breast lesions.

ADH5 is a cocktail of antibodies that specifically target myoepithelial and epithelial antigens. It employs a dual chromogenic detection system, with a brown chromogen used for nuclear p63 and basal cytokeratins CK5 and CK14 and a red chromogen used for luminal cytokeratins CK7 and CK18. This staining technique has proven particularly valuable when the amount of biopsy material containing carcinoma is limited. By using ADH5, pathologists can better identify the presence of carcinoma in biopsy samples [8].

However, the sensitivity of ADH5 in identifying low-grade carcinomas has not been thoroughly investigated. When suspicious areas of microinvasive carcinoma are present, it becomes crucial to colocalize all five antibody signals on a single slide. This is because these areas are often small and may not be easily detected on deeper cuts of tissue blocks. Achieving such colocalization allows for a more accurate assessment of the presence and extent of microinvasion. After the diagnosis of invasive breast carcinoma, it is recommended to conduct hormone receptor and HER2 testing on both primary and recurrent/metastatic tumors. These tests provide essential information for prognosis and guide treatment decisions. Hormone receptor status and HER2 expression are important prognostic and predictive markers in breast cancer.

We often use the ADH5 cocktail stain in our practice to address various diagnostic challenges. It aids in identifying microinvasion and foci of invasive carcinoma, distinguishing atypical ductal hyperplasia from hyperplasia of the usual type, and differentiating basal phenotypes in triple-negative carcinomas.

During our routine use of the ADH5 cocktail stain, an unexpected staining pattern was observed in low-grade infiltrating duct carcinoma (NOS) cases with tubular features and more so in tubular carcinomas. This observation prompted further investigation into the matter.

We reviewed the ADH5 cocktail staining of low-grade invasive breast carcinoma (NOS) and tubular carcinomas to address this issue. We aimed to compare the ADH5 cocktail staining with the staining results obtained using other individual myoepithelial markers, such as p63 and SMMH. The objective was to assess the ease of interpretation and the ability of these staining techniques to provide valuable diagnostic information.

By evaluating and comparing these staining techniques, we aimed to enhance our understanding of their performance in diagnosing and characterizing low-grade invasive breast carcinomas. This knowledge can contribute to improved diagnostic accuracy and aid in proper management of patients with breast cancer.

## 2. Materials and Methods

### 2.1. Cases

After obtaining approval from the institutional review board, we identified consecutive cases of breast lumpectomies and mastectomies conducted within our institution. Specifically, we focused on patients diagnosed with low-grade invasive breast carcinoma (NOS) with tubular features and tubular carcinomas from January 2017 to December 2021. A total of 44 cases were included in this study, and they were selected based on their consecutive occurrence.

To ensure the accuracy of the diagnoses, the study’s authors, including our specialized breast pathologists, conducted a thorough review of all relevant sections stained with hematoxylin and eosin (H&E), as well as immunohistochemistry (IHC). This comprehensive review confirmed the diagnoses of all patients included in the study. Out of the selected cases, there were 28 lumpectomies and 16 mastectomies, which provided a diverse range of samples for analysis.

Alongside the histological review, we gathered clinicopathological information specific to each case from the corresponding pathology reports. This information encompassed a range of relevant clinical and pathological details, including patient demographics, tumor characteristics, and other pertinent factors.

Our aim was to conduct a rigorous and informative investigation into the staining patterns of low-grade invasive breast carcinoma (NOS) with tubular features by assembling this well-defined cohort of cases and collecting comprehensive clinicopathological information. The data obtained from this study would contribute to a better understanding of these specific subtypes of breast cancer and their diagnostic implications, ultimately leading to improved patient care and treatment decisions.

### 2.2. Immunohistochemistry

Unstained slides were prepared from the appropriate paraffin block of each case and stained with ADH5 cocktail (CK5/14, P63, and CK7/18) and smooth muscle myosin heavy chain (SMMH) using the Ventana Benchmark Ultra System after antigen retrieval was applied. For the ADH5 cocktail: Predilute primary antibody (AVI3204) was applied to tissue sections for 32 min at 37 °C, followed by the use of an Ultraview DAB detection kit. Then, denaturation was conducted for 4 min at 90 °F. This was followed by the use of the Roche Ultraview universal alkaline phosphatase red detection kit. Slides were then counterstained with hematoxylin, dehydrated (air dry and xylene), and cover-slipped. Normal breast ducts and lobules were used as a positive control. For SMMH staining, after sections were backed and deparaffinized, a primary antibody, SMMS-1, was applied for 16 min at 37 °C. Antigens were visualized with an I-View DAB detection kit. Slides were then counterstained with hematoxylin, dehydrated (air dry and xylene), and cover-slipped. A normal large intestine was used as a positive control. Table 1 shows each antibody’s clone, dilution, pretreatment, and vendor.

### 2.3. Review of the Morphology

In this study, histological and immunohistochemical slides were analyzed by three authors. They examined the hematoxylin and eosin (H&E) slides along with the corresponding immunohistochemistry (IHC) slides stained with the ADH5 cocktail or single stains for P63 and SMMH. The aim of the study was to assess the staining characteristics of carcinoma cells and surrounding normal ducts.

In both intraductal/luminal epithelium and myoepithelial cells, positive staining for CK5/14 was identified as brown cytoplasmic staining. In the intraductal/luminal epithelium cells, positive staining for CK7/18 was found as red cytoplasmic staining. Furthermore, positive staining for p63 was observed in myoepithelial cells and was indicated by brown nuclear staining and cytoplasmic SMMH staining. These staining patterns were recorded and analyzed, as summarized in Table 2.

The evaluation of the immunostained slides was performed based on two main criteria. The first criterion focused on the ease of visual interpretation, which relied on the distribution of chromogen and antibodies. This assessment considered the ease with which pathologists could observe and interpret the staining patterns. The second criterion focused on the amount of diagnostic information provided by the staining patterns. The authors recorded both expected and unexpected patterns, indicating the extent to which the staining helped make accurate diagnoses and provide valuable diagnostic information.

Considering these criteria, the authors aimed to evaluate and compare the staining characteristics of the ADH5 cocktail versus individual stains for P63 and SMMH. This analysis aimed to provide insights into the interpretative ease and diagnostic information provided by each staining method, potentially guiding pathologists in their clinical practice and improving the accuracy of breast cancer diagnoses.

## 3. Results

### 3.1. Characteristics of the Specimen and Its Morphology

An overview of the cases analyzed in the study is provided in Table 3, which summarizes our findings. Among the 44 cases included in the analysis, 38 were classified as tubular carcinomas, while the remaining six were categorized as low-grade invasive breast carcinoma (NOS) with tubular features. These classifications were based on the examination of histological slides.

Among the 44 invasive carcinomas, 16 cases were identified in mastectomy specimens, meaning that these cases involved the surgical removal of the entire breast. On the other hand, the remaining 28 cases were observed in lumpectomy specimens, which involved the removal of only a part of the breast tissue. It is worth noting that in this study, we did not further evaluate the histological characteristics of in situ carcinoma. As a result, the focus was primarily on analyzing the staining features of invasive carcinomas.

By specifying the number of cases, the types of carcinomas included in the study, and the information about the specimen types (mastectomy vs. lumpectomy), a clear overview of the study population was provided. This information established the context for the subsequent analyses and conclusions drawn from the study.

### 3.2. Immunohistochemistry

A total of 44 cases consisting of invasive breast carcinoma samples were analyzed. Among these cases, four had been stained with the ADH5 cocktail at the time of diagnosis, while the remaining 40 cases were retrospectively stained for this study. This study’s aim was to assess the usefulness and interpretability of the ADH5 multiplex stain in invasive carcinoma cases that were previously diagnosed.

**Figure 1:**
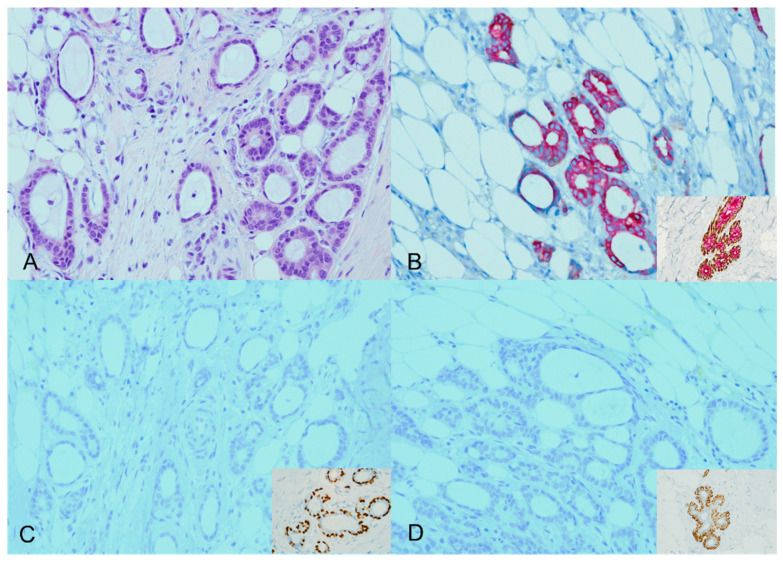
ADH5 stain showing the expected pattern of staining. An invasive carcinoma showing a glandular growth pattern and a low nuclear grade with desmoplastic stroma (H&E) ((**A**), ×20). The cytokeratin cocktail stain confirms the presence of invasive ductal carcinoma (CK7/18 red); inset for the positive internal control ((**B**), ×20). The absence of corresponding myoepithelial stains (loss of brown P63 and CK5/14) confirms the invasive carcinoma; inset for the positive internal control ((**C**,**D**), ×20).

Four of the 44 cases analyzed (9.1%) showed an expected staining pattern for ADH5. Specifically, two cases were identified as tubular carcinomas, and the other two cases were classified as low-grade infiltrating duct carcinoma (NOS) cases with tubular features. In the expected staining patterns, brown staining (representing P63 and CK5/14) was lost around the invasive glands, accompanied by diffuse red staining (representing CK7/18). This staining pattern was consistent with the expected results Figure 1.

**Figure 2:**
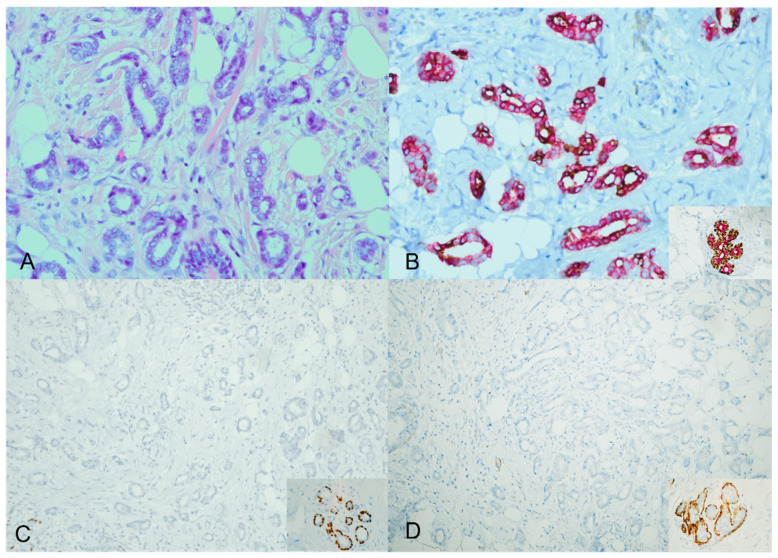
ADH5 stain showing an unexpected pattern of staining. An invasive carcinoma showing a glandular growth pattern and a low nuclear grade with desmoplastic stroma (H&E) ((**A**), ×20). The cytokeratin cocktail shows the invasive ductal carcinoma foci staining with mixed cytoplasmic brown and red; inset for the positive internal control ((**B**), ×20). The corresponding additional single myoepithelial stains confirm the loss of myoepithelial stains (loss of P63 and SMMH); inset for the positive internal control ((**C**,**D**), ×20).

However, the majority of the cases—specifically, 40 out of 44 (91%)—displayed an unexpected staining pattern. A mixture of cytoplasmic brown and red staining characterized this pattern. The ADH5 cocktail stain did not produce the distinct brown and red staining separation observed in the expected way. Instead, both cytoplasmic brown and red staining were simultaneously present Figure 2.

Additionally, we performed more staining on a subset of cases to assess the expression of 34betaE12 (high-molecular-weight cytokeratin) in expected and unexpected ADH5 staining patterns. The results showed that 34betaE12 was lost in low-grade infiltrating duct carcinomas with an expected ADH5 staining pattern. In contrast, 34betaE12 was positive in the subset with an unexpected staining pattern.

In all 44 cases (100%), there was negative staining for the individual myoepithelial markers P63 and SMMH around the invasive foci. This meant that the myoepithelial cells surrounding the invasive areas did not exhibit positive staining for P63 and SMMH, as seen in the staining results.

By providing these detailed findings, we shed light on the performance and outcomes of the ADH5 cocktail stain in the examined cases. The unexpected staining patterns observed in most cases underscore the need for further investigation and the consideration of alternative staining techniques or markers to accurately assess the presence of invasive carcinoma and differentiate it from non-invasive lesions.

## 4. Discussion

Breast ductal lesions encompass a range of conditions, including benign (usual ductal hyperplasia), borderline (atypical ductal hyperplasia), preinvasive (ductal carcinoma in situ), and invasive (invasive ductal carcinoma) conditions. Distinguishing among these conditions is crucial for distinct management approaches [9,10].

Molecular and immunohistochemical studies have contributed to classifying breast cancer based on gene expression and protein markers. The “basal-like” subtype refers to tumors with high gene expression associated with basal epithelial cells in normal mammary glands, such as CK5/6, CK14, CK15, and CK17. These tumors typically fall into the triple-negative category, lacking expression of estrogen receptor (ER), progesterone receptor (PR), and human epidermal growth factor receptor 2 (HER2). On the other hand, the luminal subtypes include luminal A (ER+ [strong], PR+, HER2−) and luminal B (ER+ [weak/moderate], PR−, sometimes HER2+). Luminal cytokeratins associated with these subtypes are CK7/8, 18, and 19 [5,11,12,13,14].

Basal-like cancers often exhibit high-grade morphology, while luminal A breast cancers tend to be of a low grade with slower growth rates and a more favorable prognosis compared to luminal B cancers [5]. Immunohistochemical markers, such as P63, are used to assess the presence or absence of myoepithelial cells in challenging cases of invasive carcinoma. Combining the nuclear p63 stain with a cytoplasmic stain, such as high molecular weight cytokeratins (CKs), can provide a more precise observation of attenuated myoepithelial cells [3].

Previously, the range of available immunohistochemical stains was limited, but now, several markers, including alpha-SMA, SMMH, Caldesmon, S100, and P63, can detect myoepithelial cells.

The limited availability of lesional tissue in biopsy materials is a common challenge. After proving the diagnosis of breast carcinoma, the remaining tissue in the biopsy is needed to assess further biomarkers, such as estrogen/progesterone receptors and HER2/neu expression, which guide clinical management and treatment decisions [15].

The ADH5 cocktail stain, which consists of p63/CK7/18/CK5/14 antibodies, is valuable in breast pathology diagnostics, particularly for assessing potential invasion and distinguishing between typical hyperplastic and atypical ductal epithelial proliferation. It has proven significant diagnostic usefulness, especially in detecting minute foci of invasion [16]. Multiplex immunohistochemistry—combining multiple antibodies in a single staining procedure—shows promise in enhancing diagnostic capabilities in breast pathology and other organ systems [17,18,19].

However, using multiplex stains presents challenges, including technical issues and interpretative faults [17,20,21]. Several studies have investigated the tissue integrity, staining quality, and ease of interpretation of different multiplex stains in breast pathology, such as the Minimal Carcinoma triple stain (MC), Breast Triple Stain (BTS), and LC/DC Breast Cocktail (LCDC). These stains are designed to differentiate intraepithelial ductal proliferation, clarify the presence and extent of invasive carcinoma, or classify ductal or lobular proliferation [15].

This study aimed to evaluate the diagnostic utility of the ADH5 cocktail stain in low-grade ductal carcinomas by assessing the visual ease of interpretation based on the chromogen and antibody composition. After reviewing a positive internal control, which indicated proper processing and staining of all samples, unexpected staining patterns were seen in 91% (40 out of 44) of cases. These cases exhibited a mixture of cytoplasmic brown and cytoplasmic red staining instead of the expected pattern. While unexpected staining patterns can occur in multiplex stains, it is crucial to be aware of these potential pitfalls [6]. This highlights the importance of not relying solely on immunohistochemistry for diagnosis and emphasizes the need for careful histopathological evaluation [21].

We additionally performed staining on a subset of cases to assess the expression of 34betaE12 in expected and unexpected ADH5 staining patterns. The results showed that 34betaE12 was lost in cases with an “expected” ADH5 staining pattern and was positive in the unexpected cases. The positive staining of 34betaE12 in low-grade invasive carcinoma that is stained unexpectedly by ADH5 may be attributed to alterations in cytokeratin expression or antigen accessibility within the tumor microenvironment. Although 34betaE12 expression has been investigated in several cancers, including breast cancer, its role is unclear. Further molecular and cellular studies in a larger cohort of cases could shed light on the underlying reasons for these findings.

## 5. Conclusions

The unexpected ADH5 staining pattern observed in invasive carcinomas and low-grade cytological atypia may lead to a misdiagnosis due to the resemblance to benign glands. Therefore, users of multiplex stains should exercise careful interpretation when encountering low-grade invasive ductal carcinoma cases. These findings highlight the importance of integrating immunohistochemistry with comprehensive histopathological evaluation to ensure an accurate breast pathology diagnosis.

Future studies can delve into the underlying causes of unexpected staining patterns observed in low-grade ductal carcinomas by exploring the interactions among antibodies in multiplex stains, tumor heterogeneity, and the influence of tissue processing techniques. Understanding these factors can guide improvements in staining protocols and interpretation criteria. Additionally, efforts should focus on developing standardized protocols for multiplex staining in breast pathology by optimize antibody combinations, staining conditions, and interpretation criteria to ensure consistency and reproducibility across different laboratories and institutions.

Pursuing these research directions can enhance diagnostic accuracy, improve treatment decision making, and advance our understanding of breast ductal lesions, ultimately leading to improved patient outcomes.

## Figures and Tables

**Table 1:** Clone, dilution, pretreatment, and vendor of the antibodies used for the stains that we applied.

Antibody	Clone	Pretreatment	Isotype	Type	Vendor
Anti-CK 5/14	34βE12	Ultra CC1 standard (64 min) at 95 °C	IgG1/kappa	Mouse monoclonal	Biocare medical/CA
Anti-P63	4A4		IgG2a/kappa	Mouse monoclonal	
Anti-CK7	BC1		IgG	Rabbit monoclonal	
Anti CK18	5D3		IgG1	Mouse monoclonal	
Anti-SMMH	SMMS-1	CC1 (36 min) at 95 °C	IgG1/kappa	Mouse monoclonal	Cell Marque/Sigma-Aldrich company/CA
Anti-CK HMW	34βE12	CC1 (46 min) at 95 °C	IgG	Mouse monoclonal	Roche/Venatna

**Table 2:** The immunohistochemical stains that we used.

Antibody	Staining	Cell Type	Stain Localization
Anti-CK 5/14	DAB Brown	Myoepithelial/Luminal Basal Phenotype	Cytoplasmic
Anti-P63	DAB Brown	Myoepithelial cells	Nuclear
Anti-CK 7/18	FR Red	Luminal epithelium	Cytoplasmic
Anti-SMMH	DAB Brown	Myoepithelial cells	Cytoplasmic
Anti-CK HMW	DAB Brown	Myoepithelial/Luminal Basal Phenotype	Cytoplasmic

**Table 3:** Characteristics of breast carcinomas highlighted with the ADH5 stain.

Clinicopathologic Characteristics	No.	%
Median age:		
- 40–49	7	15.9%
- 50–59	12	27.2%
- 60–69	8	18.1%
- 70–79	14	31.8%
- 80–85	3	6.8%
Specimen type:		
- Lumpectomy	28	63.6%
- Total mastectomy	16	36.3%
Specimen laterality:		
- Right	17	38.6%
- Left	27	61.3%
Size of the lesion:		
- 0.1–0.9 cm	22	50%
- 1.0–1.9 cm	17	38.6%
- 2.0–2.9 cm	3	6.8%
- 3.0–3.5 cm	2	4.5%
ER status		
- Positive	44	100%
- Negative	0	0%
PR status		
- Positive	38	86.3%
- Negative	6	13.6%
HER2 status		
- Positive	0	0%
- Negative	44	100%
ADH5 staining		
- Expected	40	9.1%
- Unexpected	4	90.9%

Abbreviations: ER: estrogen receptor, PR: progesterone receptor, HER2: human epidermal growth factor receptor 2.

## Data Availability

All data and materials are available upon request from the corresponding author.
